# Bosentan-based, treat-to-target therapy in patients with pulmonary arterial hypertension: results from the COMPASS-3 study

**DOI:** 10.1177/2045893217741480

**Published:** 2017-10-24

**Authors:** Raymond L. Benza, Amresh Raina, Himanshu Gupta, Srinivas Murali, Annie Burden, Michael S. Zastrow, Myung H. Park, Marc A. Simon

**Affiliations:** 1 6618 Allegheny General Hospital, Pittsburgh, PA, USA; 2 University of Alabama at Birmingham, Birmingham, AL, USA; 3 Statistical Consultancy, Quanticate, Hitchin, Hertfordshire, UK; 4 17430 Former employee of Actelion Pharmaceuticals US, South San Francisco, CA, USA; 5 23534 Houston Methodist Hospital, Houston, TX, USA; 6 University of Pittsburgh Medical Center, Pittsburgh, PA, USA

**Keywords:** bosentan, cardiac magnetic resonance imaging, combination therapy, pulmonary arterial hypertension, sildenafil

## Abstract

The phase 4 COMPASS-3 study evaluated whether a singular endpoint produces clinically meaningful outcomes in patients with pulmonary arterial hypertension (PAH). The relationship between cardiac magnetic resonance imaging (cMRI)-derived parameters and right heart catheterization (RHC) measurements was also examined. In COMPASS-3 (ClinicalTrials.gov NCT00433329), 100 patients with PAH received bosentan monotherapy for 16 weeks. Patients continued monotherapy if their 6-min walk distance (6MWD) was ≥380 m, or otherwise received add-on sildenafil for an additional 12 weeks. 6MWD, RHC, and cMRI were performed at baseline, week 16, and week 28 (6MWD and cMRI). Baseline median 6MWD was 274 m and 82% of patients had WHO Functional Class III/IV. At week 16, 17% (n = 16) of remaining patients achieved the 6MWD threshold and 78 (83%) did not. In the intention-to-treat population, median 6MWD increased significantly relative to baseline (week 16 = 308 m; week 28 = 327 m; *P* < 0.001). At week 28, 9/16 (monotherapy) and 15/76 (20%; add-on sildenafil) patients met the target threshold. Baseline cMRI-derived and RHC-derived parameters showed moderate-to-strong correlations (e.g. right to left ventricular end-diastolic ratio [RVEDV:LVEDV] correlated strongly with pulmonary vascular resistance [r = +0.729, *P* < 0.0001]). cMRI-derived parameters predicted clinical worsening/decline (e.g. week 16 RVEDV:LVDEV [*P* = 0.0172]). Time to clinical worsening/decline did not differ between patients based on 6MWD threshold achievement. No unexpected safety events were reported. A substantial proportion of patients failed to achieve the goal of 380 m, regardless of treatment. Several cMRI parameters predicted clinical worsening/decline and its non-invasive nature further supports its use in future clinical trials.

## Introduction

The availability of targeted disease-specific therapies has led to improvements in exercise capacity, World Health Organization (WHO) functional class (FC), and survival in patients with pulmonary arterial hypertension (PAH).^1–4^ Despite these advances, the prognosis of patients with PAH remains poor, with a five-year survival rate of 55–57%.^4,5^

The change in 6-min walk distance (6MWD) has been widely utilized as a primary endpoint in clinical trials of PAH to gauge treatment response.^5–8^ In an observational study of 178 patients receiving PAH-specific therapy, a 6MWD ≥ 380 m was found to correlate with improved survival^5^ and, until recently, this threshold has been recommended as a therapeutic goal.^9,10^ As 6MWD can vary with patient age, height, sex, and co-morbid illness,^11^ the predictive value of specific 6MWD thresholds has been questioned;^12^ however, one-year survival estimates are consistently higher for patients who score above threshold compared with patients who score below threshold, regardless of specific threshold (i.e. <165, 165–440, and >440 m).^13^ In addition to 6MWD, disease etiology, cardiac output, and right atrial pressure also predict mortality risk in patients with PAH.^14,15^

Hemodynamic parameters, measured via right heart catheterization (RHC), are also used to measure treatment response. However, RHC is an invasive procedure, which limits the ability to acquire serial measurements.^9,16^ In contrast, cardiac magnetic resonance imaging (cMRI) is a non-invasive, high-resolution technique allowing for the visualization and direct measurement of anatomical and functional changes in the right heart (i.e. enhanced volume and pressure measurements compared with echocardiography).^9,8,17–19^ cMRI-derived parameters, such as right ventricular (RV) volumes and ejection fraction, correlate with traditional measures of functional status, including 6MWD,^20–22^ and survival.^23–27^ cMRI is becoming an important tool in the clinical study of PAH as it can provide valuable information in an accurate, reproducible, and non-invasive manner.

COMPASS-3 was an open-label, non-comparative phase IV study that evaluated whether treating a patient to a single prespecified target (6MWD ≥ 380 m) produces clinically meaningful results. The study was also designed to evaluate the utility of cMRI in assessing improved functional capacity in patients with PAH and to explore the correlation between cMRI-derived parameters and traditional assessments of patient clinical status.

## Methods

### Study design

COMPASS-3 (NCT00433329) was an open-label, exploratory phase 4 study conducted in 23 sites in the United States during 2007–2010. This study was initiated before the publication of recent guidelines which support a treatment approach that involves comprehensive assessment of patient characteristics with the goal of reducing mortality risk (treat-to-outcomes rather than treat-to-target) approach for clinical trials.^9^ Following a screening period of ≤2 weeks, treatment-naïve patients received oral twice-daily bosentan 62.5 mg for 4 weeks followed by twice-daily bosentan 125 mg (or 62.5 mg, if 125 mg was poorly tolerated) for 12 weeks (Fig. S1). Patients achieving a 6MWD ≥ 380 m at 16 weeks remained on bosentan monotherapy for an additional 12 weeks (125 mg twice daily), while those who did not would go on to receive combination therapy—beginning at week 16—with twice-daily bosentan 125 mg plus sildenafil 20 mg three times daily for an additional 12 weeks. COMPASS-3 conformed to Good Clinical Practice guidelines and Declaration of Helsinki principles. The protocol was approved by the Institutional Review Board/Independent Ethics Committee at each participating site, as described in the online supplement.

### Patients

Inclusion criteria included patients aged ≥21 years diagnosed with WHO Group I PAH who were treatment-naïve (i.e. not considered to be candidates for parenteral prostacyclins, per the discretion of the treating physician). PAH was diagnosed by RHC findings of mean pulmonary artery pressure (mPAP) ≥25 mmHg; pulmonary artery wedge pressure (PAWP) or left ventricular end diastolic pressure ≤15 mmHg; and pulmonary vascular resistance (PVR) ≥3 Wood units (WU). Baseline 6MWD entry criterion was 150–360 m. Exclusion criteria are described in the online supplement.

### Endpoints

The primary prespecified endpoint was the proportion of patients who achieved a 6MWD ≥ 380 m at 16 weeks and/or at 28 weeks. Hypothesis-generating post-hoc endpoints are described in the online supplement including the change from baseline to weeks 16 and 28 in 6MWD and percent predicted 6MWD, WHO FC, NT-pro-brain natriuretic peptide (NT-pro-BNP), RHC-related parameters (week 16 only), and cMRI-derived parameters. A final follow-up was scheduled at week 52.

### Assessments

6MWD was measured per American Thoracic Society guidelines.^28^ NT-pro-BNP was quantified at a central laboratory (Quintiles Inc.). Hemodynamic evaluations were performed with the patient in the supine position per local standard practice utilizing the internal jugular, subclavian, or femoral vein and a triple (or 4)-lumen, balloon-tipped, thermo-dilution catheter. Cardiac output was measured by either thermo-dilution, measured in triplicates with <10% differences, or the Fick principle, with the same method used for a patient throughout the study.

cMRI was performed using a 1.5-T magnet and software capable of cardiovascular imaging.^29^ cMRI variables were indexed using baseline body surface area. Bright-blood cine images were acquired using an electrocardiographic-gated steady-state free precession technique.^30^ Images were sent to a central core laboratory (University of Alabama, Birmingham, AL, USA) for analysis and interpretation. RHC and cMRI were completed within a 48-h period at baseline and week 16. An additional cMRI examination was performed at week 28.

Clinical worsening was defined as hospitalization for worsening in, or complications of, PAH, atrial septostomy, lung transplantation, initiation of parenteral prostanoids, or death between baseline and week 52. Clinical decline was defined as worsening of ≥1 WHO FC plus ≥15% decline in 6MWD between baseline and week 52.

### Statistical analyses

Efficacy and safety analyses were performed on the intention-to-treat (ITT) population, which was composed of all patients who received ≥1 dose of study drug. As this was an exploratory study, no formal statistical hypothesis testing was planned; however, *P* values were generated for illustrative purposes.

For the primary endpoint, patients who did not have a 6MWD result available (regardless of the reason) were considered non-responders and included in the denominator. At week 28, the proportion of patients who achieved a 6MWD ≥ 380 m were summarized for the ITT population and the subgroups of patients receiving bosentan monotherapy or bosentan plus sildenafil combination therapy. The differences in 6MWD and percent predicted 6MWD between the treatment groups at various time-points were compared using the Mann–Whitney test and Hodges–Lehman 95% confidence intervals (CIs). Patient demographics and disease characteristics at baseline were compared post hoc in the monotherapy and combination therapy groups using a mixed model for continuous variables and chi-square test for categorical variables.

*P* values were calculated for changes from baseline to weeks 16 and 28 for 6MWD, NT-pro-BNP, RHC-related parameters (week 16 only), and cMRI-derived parameters using the t-test for mean values and Wilcoxon rank test for median values. For proportions, 95% CIs were computed from the Clopper–Pearson (Exact) method. For mean values, 95% CIs were computed as the sample mean ± the appropriate quantile of t-distribution × the standard error. For median values, 95% CIs were computed based on a distribution-free method. As part of an exploratory analysis, the correlation between cMRI-derived parameters and traditional patient assessments at baseline and week 16 was estimated using Spearman rank-order correlation coefficients with 95% CIs and associated *P* values. Parameters examined in correlation analyses are listed in Table S1.

In a preplanned analysis, time to clinical worsening and/or decline was estimated using the Kaplan–Meier method. In a post-hoc analysis, the difference between the monotherapy and combination therapy treatment groups in the time to clinical worsening and/or decline was compared using the Wilcoxon log-rank test. The characteristics of patients who did and did not experience clinical worsening or decline were examined post-hoc. The same statistical tests used to compare the monotherapy and combination therapy treatments groups were employed.

Further comparisons between patients in the monotherapy and combination therapy groups were made using the REVEAL risk score calculator for patients with PAH.^14^ Developed using patients in the REVEAL registry, the REVEAL risk score calculator predicts patient 12-month survival based on demographic, clinical, and hemodynamic variables. Comparisons between groups were made using an independent t-test with *P* < 0.05 considered statistically significant.

Predictors of clinical worsening and/or decline were explored post-hoc using univariable and multivariable logistic regression. All parameters with *P* ≤ 0.10 in the univariable analyses were included in the multivariable analyses. The multivariable models were manually stepwise-reduced to identify groups of non-collinear parameters significantly predictive of clinical worsening and/or decline. All statistical analyses were performed using SAS® version 8.2 or later.

## Results

### Patient disposition and characteristics

**Fig. 1.**
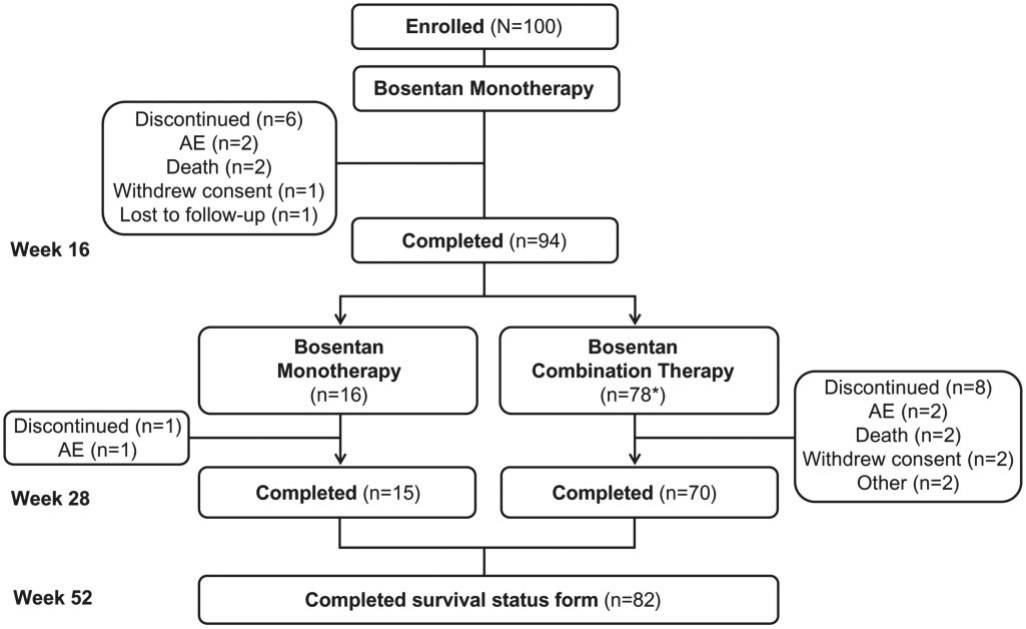
Patient disposition. *Two patients withdrew consent before dosing. Thus, only 76 patients received combination treatment. AE, adverse event.

One hundred patients were enrolled and included in the ITT population (Fig. 1). Of these, 94% completed the 16-week monotherapy phase and 85% completed 28 weeks of treatment. Baseline demographics for the ITT population are summarized in Table 1 and functional and biomarker characteristics at baseline are shown in Table 2. Baseline hemodynamics were consistent with advanced PAH (Table 3) and cMRI-derived parameters at baseline confirm the enrollment of a population with adverse RV remodeling (Table 4). cMRI data were excluded in three patients due to the poor quality or inadequate acquisition of results.

**Table 1.** Patient demographics and disease characteristics at baseline.

	Patients (n = 100)
Median age (range), years	57.5 (21–84)
Female, n (%)	82 (82)
Median BMI (range), kg/m^2^*	29.3 (14.5–47.4)
Race, n (%)	
White	81 (81)
Black, African American, or African heritage	18 (18)
Other	1 (1)
PAH etiology, n (%)	
Idiopathic	56 (56)
Associated	40 (40)
Connective tissue disease	26 (26)
Other^†^	13 (13)
Congenital heart disease	1 (1)
Familial	4 (4)
Median time since diagnosis (range), years	0.12 (0–20.92)^‡^

* n = 99.

† Not specified.

‡ One female patient (aged 58 years) was diagnosed with idiopathic PAH 20.94 years before study start. The next longest time since diagnosis was 4.87 years.

BMI, body mass index; PAH, pulmonary arterial hypertension.

**Table 2.** Clinical, functional, and biomarker parameters at baseline, week 16, and week 28.

	Baseline (n = 100)	Week 16 (n = 100)	Week 28 (n = 100)
Median 6MWD (range), m	n = 100 273.6 (152–360)	n = 94 307.9 (24–517)	n = 84 326.5 (45–640)
Median change from baseline, m (range)	–	n = 94 27.6 (−220 – 195) *P* < 0.001	n = 84 50.0 (−230 – 430) *P* < 0.001
Median % predicted 6MWD, % (range)*	n = 99 53.2 (28.8–97.1)	n = 93 58.7 (5.1–125.5)	n = 84 64.8 (10.6–140.4)
Median change from baseline, % (range)	–	4.66 (−48.84 – 45.39) *P* = 0.002	9.54 (−46.92 – 92.39) *P* < 0.001
WHO FC, n (%)	n = 100	n = 93	n = 84
I	1 (1)^†^	6 (6)	6 (7)
II	17 (17)	25 (27)	34 (40)
III	79 (79)	59 (63)	42 (50)
IV	3 (3)	3 (3)	2 (2)
Missing	0	7	16
Median NT-pro-BNP, pg/mL (range)^‡^	n = 88^§^ 988.5 (20–52,766)	n = 90 702.0 (35–16,419)	n = 83 566.0 (33–28,276)
Median change from baseline, pg/mL (range)	–	n = 80 −73.0 (−14,611 – 10,902) *P* = 0.055	n = 74 −73.0 (−14,685 – 5,868) *P* = 0.008

* The predicted 6MWD was calculated for men as follows: 7.57 × height (cm) – 5.02 × age (years) – 1.76 × weight (kg) – 309 m. The predicted 6MWD was calculated for women as follows: 2.11 × height (cm) – 5.78 × age (years) – 2.29 × weight (kg) + 667 m.

† The patient was male, aged 28 years, and his 6MWD at baseline was 352 m (% predicted = 47%).

‡ The median value is presented due to the skewed distribution of these data.

§ The vials for 12 samples were broken during transport to or at the central laboratory.

6MWD, 6-minute walk distance; NT-pro-BNP, N-terminal pro-B-type natriuretic peptide; WHO FC, World Health Organization functional class.

**Table 3.** RHC-derived hemodynamic parameters.

	Baseline (n = 100)	Week 16 (n = 100)	Mean change from baseline (SD) (n = 100)	*P* value
Mean mRAP, mmHg (SD)	n = 99 9.7 (5.73)	n = 92 9.3 (6.70)	n = 91 −0.3 (7.03)	0.733
Mean mPAP, mmHg (SD)	n = 100 46.1 (14.04)	n = 92 41.5 (14.09)	n = 92 −4.3 (9.09)	<0.001
Mean PVR, Wood units (SD)	n = 99 9.8 (5.73)	n = 89 7.4 (4.73)	n = 88 −2.1 (2.94)	<0.001
Mean PVR index, WU/m^2^ (SD)	n = 98 5.5 (3.43)	n = 88 4.2 (2.86)	n = 87 −1.2 (1.70)	<0.001
Mean SV:PP, (L/min)/(bpm/mmHg) (SD)	n = 95 1.5 (0.74)	n = 90 1.6 (0.78)	n = 85 0.2 (0.93)	0.096
Mean SVR:PVR (SD)	n = 97 2.7 (1.44)	n = 89 3.6 (3.78)	n = 86 0.8 (3.30)	0.02
Mean SVO_2_, % (SD)	n = 85 62.9 (9.02)	n = 78 65.1 (11.12)	n = 75 2.4 (9.54)	0.03
Mean cardiac output, L/min (SD)	n = 100 4.3 (1.42)	n = 91 4.6 (1.43)	n = 91 0.4 (0.98)	<0.001
Mean cardiac index, L/min/m^2^ (SD)	n = 99 2.3 (0.69)	n = 90 2.5 (0.67)	n = 90 0.2 (0.54)	<0.001

mPAP, mean pulmonary artery pressure; mRAP, mean right artery pressure; PP, pulse pressure; PVR, pulmonary vascular resistance; SD, standard deviation; SV, stroke volume; SVO_2_, mixed venous oxygen saturation; SVR, systemic vascular resistance.

**Table 4.** cMRI-derived parameters.

	Baseline (n = 100)	Week 16 (n = 100)	Mean change from baseline (SD) (n = 100)	*P* value
Mean RVEDV index, mL/m^2^* (SD)	n = 95 102.7 (37.27)	n = 87 102.8 (36.44)	n = 83 −0.8 (19.62)	0.702
Mean RVESV index, mL/m^2^* (SD)	n = 95 61.5 (35.30)^†^	n = 87 58.1 (33.97)	n = 83 −4.4 (15.46)	0.012
Mean RVEF, % (SD)	n = 96 43.4 (14.96)	n = 88 46.4 (15.13)	n = 84 3.1 (8.47)	0.001
Mean LVEDV index, mL/m^2^* (SD)	n = 95 60.4 (18.17)	n = 89 63.3 (17.90)	n = 85 3.5 (10.45)	0.003
Mean LVESV index, mL/m^2^* (SD)	n = 95 23.7 (10.33)	n = 89 21.8 (9.15)	n = 85 −1.2 (6.41)	0.085
Mean LVEF, % (SD)	n = 96 60.9 (11.19)	n = 90 65.4 (9.53)	n = 86 4.0 (10.04)	<0.001
Mean RV mass index, g/m^2^* (SD)	n = 94 28.8 (12.37)	n = 86 30.2 (13.41)	n = 82 1.1 (5.89)	0.084
Mean RV mass:LV mass ratio (SD)	n = 95 0.7 (0.31)	n = 87 0.7 (0.31)	n = 83 0.0 (0.16)	0.945
Mean RVEDV:LVEDV ratio (SD)	n = 96 1.9 (0.96)	n = 88 1.8 (0.81)	n = 84 −0.2 (0.37)	<0.001
Mean RVESV:LVESV ratio (SD)	n = 96 3.0 (1.86)	n = 88 2.9 (1.76)	n = 84 −0.2 (1.19)	0.205
Mean stroke volume index, mL/m^2^* (SD)	n = 95 36.7 (12.33)	n = 89 41.4 (12.41)	n = 85 4.7 (9.68)	<0.001

All data are mean (SD).

* Indexed variables were computed using baseline body surface area values.

† Value rounded from “0.01.”

LV, left ventricle; LVEDV, left ventricular end diastolic volume; LVEF, left ventricular ejection fraction; LVESV, left ventricular end systolic volume; RV, right ventricle; RVEDV, right ventricular end diastolic volume; RVEF, right ventricular ejection fraction; RVESV, right ventricular end systolic volume.

### 6MWD threshold achievement

In the ITT population, 31 patients (mean age = 51.1 years, standard deviation [SD] = 14.2 years) achieved the primary endpoint of a 6MWD ≥ 380 m (n = 16 at 16 weeks and/or n = 15 at 28 weeks) (Table S2). Of the 94 patients who completed 16 weeks of treatment, 16 achieved a 6MWD ≥ 380 m and continued with bosentan monotherapy. Of the 78 patients who did not achieve the 6MWD threshold at week 16, 76 received combination therapy with bosentan plus sildenafil for an additional 12 weeks; two patients withdrew consent before receiving combination therapy. In total, 24/92 (26%) of patients at week 28 achieved the 6MWD target. At all time-points, 6MWD was significantly greater in the cohort of patients who remained on monotherapy vs. combination therapy (see Table S3). At week 28, 9/16 patients who remained on monotherapy maintained a 6MWD ≥ 380 m and 15/76 patients who went on to receive combination therapy achieved the target threshold. When comparing the actual distance walked in relation to the percent predicted 6MWD, patients who remained on monotherapy had significantly higher median percent predicted 6MWD compared with the combination therapy group at week 16 (71% vs. 58%, *P* < 0.0001) and week 28 (69% vs. 61%, *P* = 0.0195).

Relative to the monotherapy group, a significantly greater proportion of patients who required combination therapy were women (87% vs. 63%, *P* = 0.0196) and were WHO FC III or IV (88% vs. 56%, *P* = 0.0073) (see Table S4). Patients who received combination therapy also had a significantly lower baseline 6MWD; the median difference for the monotherapy and combination therapy subgroups was 64.2 (95% CI = 27.1–89.0, *P* = 0.0004). No other baseline demographic, laboratory (including NT pro-BNP), or functional parameter (including percent predicted 6MWD) differed between the treatment groups. Patients on combination therapy had significantly lower baseline mean right atrial pressure (mRAP) (8.99 vs. 12.94 mmHg, *P* = 0.0137), significantly higher baseline cardiac index (2.38 vs. 2.02 L/min/m^2^, *P* = 0.0270), significantly higher cardiac output (4.40 vs. 3.66 L/min; *P* = 0.0487), and significantly higher mixed venous oxygen saturation (SVO_2_) (64.04% vs. 58.40%; *P* = 0.0348) relative to patients who remained on monotherapy. No other hemodynamic parameter or cMRI-derived variable at baseline differed between groups.

### Changes from baseline

#### ITT population

In the ITT population, median 6MWD increased significantly from 274 m at baseline to 308 m at week 16 (*P* < 0.001) and 327 meters at week 28 (*P* < 0.001) (Table 2). The median percent predicted 6MWD increased from 53.2% at baseline to 64.8% at week 28 (*P* < 0.001). The proportion of patients in WHO FC III decreased, while the proportions of patients in WHO FC I or II increased (Table 2). Among 84 patients with an assessment at both baseline and week 28, 30 improved by ≥1 WHO FC and two patients deteriorated by ≥1 WHO FC. NT-pro-BNP decreased significantly from a median of 988.5 pg/mL at baseline to 566.0 pg/mL at week 28 (*P* = 0.008), but not at week 16 (702.0 pg/mL, *P* = 0.055) (Table 2).

Following 16 weeks in the ITT population, significant decreases in mPAP (−4.3 mm Hg, *P* < 0.001), PVR (−2.1 WU, *P* < 0.001), and PVR index (−1.2 WU/m^2^, *P* < 0.001) and significant increases in cardiac output (+0.4 L/min, *P* < 0.001), cardiac index (+0.2 L/min/m^2^, *P* < 0.001), SVO_2_ (+2.4%, *P* = 0.03), and systemic vascular resistance (SVR):PVR ratio (+0.8, *P* = 0.02) were seen relative to baseline (Table 3). Between baseline and week 16, there was a significant reduction in right ventricular end systolic volume (RVESV) index (−4.4 mL/m^2^, *P* = 0.012), and an increase in right ventricular ejection fraction (RVEF) (+3.1%, *P* = 0.001) and stroke volume index (+4.7 mL/m^2^, *P* < 0.001) (Table 4). Right ventricular end diastolic volume: left ventricular end diastolic volume ratio (RVEDV:LVEDV) decreased significantly (−0.2, *P* < 0.001) between baseline and week 16, which was associated with a significant improvement in left ventricle filling (LVEDV index = +3.5 mL/m^2^, *P* = 0.003) and left ventricular ejection fraction (LVEF = +4.0%, *P* < 0.001).

Comparisons between monotherapy and combination therapy groups at weeks 16 and 28 are reported in the online supplement.

### Correlation of cMRI-derived parameters

Baseline cMRI-derived variables were correlated with RHC-derived parameters acquired at baseline. RVEDV:LVEDV correlated strongly with PVR (r = +0.729, *P* < 0.0001), mPAP (r = +0.717, *P* < 0.0001), PVR index (r = +0.704, *P* < 0.0001), and there was a low but significant correlation with mRAP (r = +0.360, *P* = 0.0003). In addition, RVEDV:LVEDV correlated inversely and strongly with SVR:PVR ratio (SVR:PVR; r = −0.778, *P* < 0.0001), and weakly with cardiac index (r = −0.393, *P* < 0.0001). RVEDV index correlations were moderate with mPAP (r = +0.507, *P* < 0.0001), PVR (r = +0.473, *P* < 0.0001), and low with PVR index (r = +0.460, *P* < 0.0001) and mRAP (r = +0.367, *P* = 0.0002). RVEDV index was inversely correlated with SVR:PVR (r = −0.581, *P* < 0.0001) and SVO_2_ (r = −0.310, *P* = 0.0049). RVEF was positively correlated with SVR:PVR (r = +0.635, *P* < 0.0001), inversely correlated with PVR (r = −0.540, *P* < 0.0001), PVR index (r = −0.499, *P* < 0.0001), and mPAP (r = −0.553, *P* < 0.0001), and had low correlations with cardiac output (r = +0.279, *P* = 0.0056), cardiac index (r = +0.295, *P* = 0.0035), SVO_2_ (r = +0.398, *P* = 0.0002), and mRAP (r = −0.373, *P* = 0.0002). Correlations between 6MWD and cMRI parameters are reported in the online supplement.

### Time to clinical worsening and/or decline

Twenty-two patients (22%) in the ITT population experienced clinical worsening and/or decline, with ten events occurring between baseline and week 16, six between weeks 16 and 28, and six between weeks 28 and 52. Because of the low number of clinical events, it was not possible to derive time-to-event estimates.

**Fig. 2.**
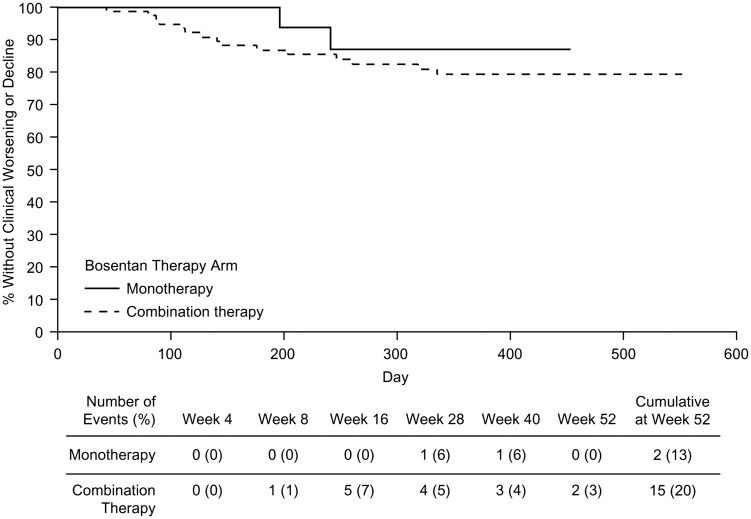
Kaplan–Meier plot of time to clinical worsening and/or clinical decline.

By week 52, 13% of patients in the monotherapy group and 20% in the combination therapy group experienced clinical worsening and/or decline (Fig. 2). There was no significant difference between the monotherapy and combination therapy treatment arms in terms of the time to clinical worsening and/or decline (*P* = 0.475). To examine underlying differences between the monotherapy and combination therapy groups, risk scores were calculated using the REVEAL registry risk score calculator. Mean (SD) risk score between monotherapy and combination therapy groups at baseline (7.2 [1.56] vs. 7.9 [1.25], *P* = 0.0609) borders statistical significance. When calculated at week 16 (when the decision was made to remain on monotherapy [6MWD ≥ 380 m] or switch to combination therapy [6MWD < 380 m]), patients in the combination therapy group had a significantly higher mean (SD) risk score compared with patients who remained on monotherapy (7.8 [1.81] vs. 6.8 [1.22], *P* = 0.0053), indicating a high burden of illness in the combination group.

Additional results comparing patients who did and did not experience clinical worsening and/or decline are reported in the online supplement.

### Predictors of clinical worsening and/or decline

**Fig. 3.**
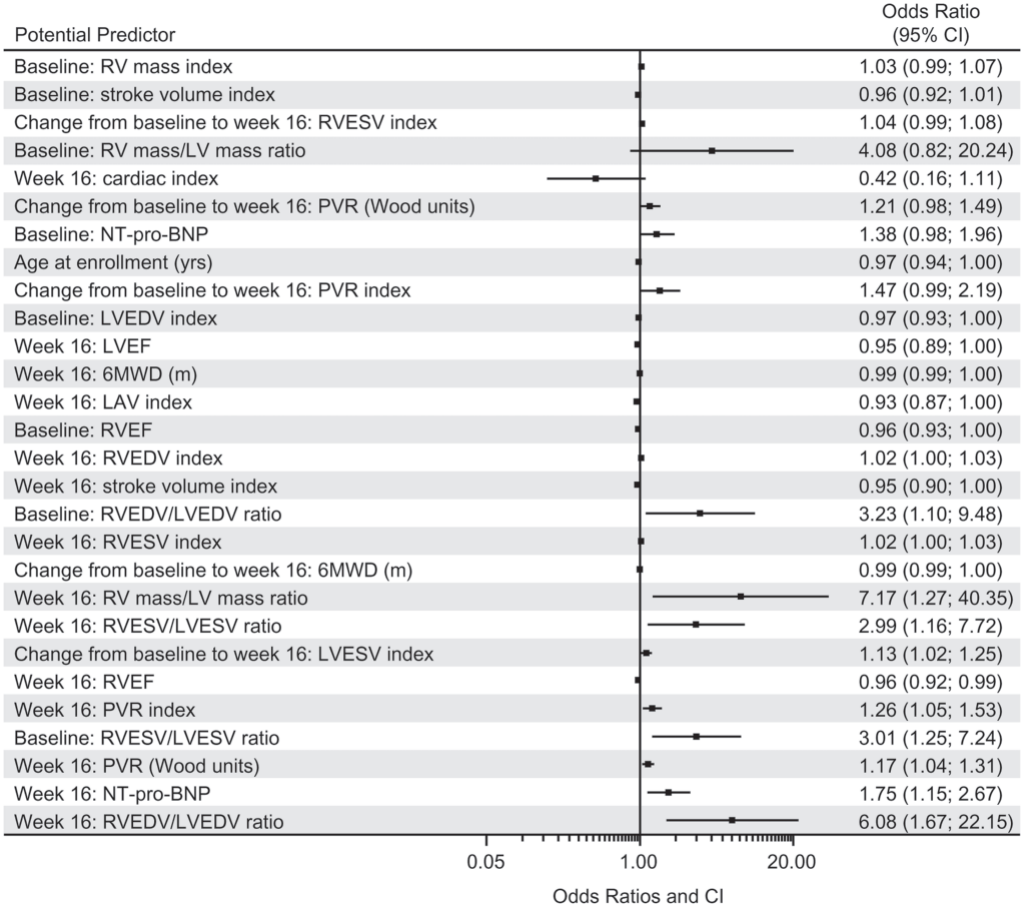
ORs from univariable analyses of baseline parameters for clinical worsening and/or decline. 6MWD, 6-min walk distance; CI, confidence interval; LAV, left atrial volume; LV, left ventricular; LVEDV, left ventricular end diastolic volume; LVEF, left ventricular ejection fraction; LVESV, left ventricular end systolic volume; NT-pro-BNP, N-terminal pro-B-type natriuretic peptide; PVR, pulmonary vascular resistance; RV, right ventricular; RVEDV, right ventricular end diastolic volume; RVEF, right ventricular ejection fraction; RVESV, right ventricular end systolic volume.

**Fig. 4.**
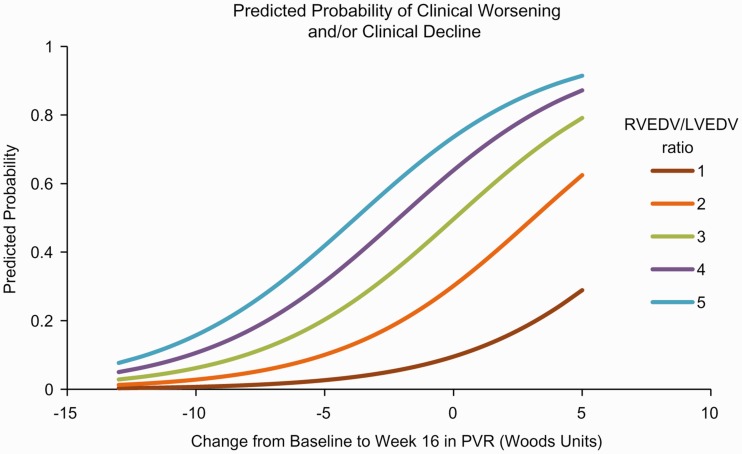
Competing outcomes plot.

Several parameters were found to be independently predictive of clinical worsening and/or decline in the univariable models. Clinical worsening and/or decline was predicted by baseline measurements of RVEF (*P* = 0.0360) and RVESV:LVESV (*P* = 0.0322). Predictors of clinical worsening and/or decline at week 16 included 6MWD (*P* = 0.0417), NT-pro-BNP (*P* = 0.0183), PVR (*P* = 0.0088), PVR index (*P* = 0.0153), RVEF (*P* = 0.0156), RVEDV index (*P* = 0.0348), RVEDV:LVEDV (*P* = 0.0172), RVESV index (*P* = 0.0291), right ventricle mass:left ventricle mass (*P* = 0.0255), left atrial volume index (*P* = 0.0376), stroke volume index (*P* = 0.0329). The change from baseline to week 16 in 6MWD (*P* = 0.0283) and LVESV index (0.0208) were also predictive of clinical worsening and/or decline. A list of all parameters predictive of clinical worsening and/or decline are provided in Table S5. Odds ratios (ORs) determined from univariable analyses are shown in Fig. 3. Three multivariable models (see online supplementary material) were generated before the final model was derived. The final multivariable model investigated the combination of a baseline cMRI-derived parameter with the changes from baseline to week 16 in PVR and 6MWD. Because of the collinearity between baseline cMRI parameters, only one could be included in the multivariable model. The best statistical model given in the equation below included the natural logarithm of baseline RVEDV:LVEDV and the change from baseline to week 16 in PVR in WU (see Table S6), which yielded the following equation for the predicted probability of clinical worsening and/or decline and is illustrated in a competing outcomes plot (Fig. 4).

$$p=\frac{\text{exp}(-2.250+2.034*{\text{log}}_{n}(\frac{\mathrm{RVEDV}}{\mathrm{LVEDV}})+0.270*\mathrm{CFBinPVR})}{1+\text{exp}(-2.250+2.034*{\text{log}}_{n}(\frac{\mathrm{RVEDV}}{\mathrm{LVEDV}})+0.270*\mathrm{CFBinPVR})}$$

where $(\frac{\mathrm{RVEDV}}{\mathrm{LVEDV}})$ is the ratio of right to left ventricular end diastolic volume at baseline, and CFB in PVR is the change from baseline to week 16 in PVR (in WU).

### Safety

No unexpected safety events were reported. Safety outcomes are summarized in Table 5 and described in detail in the online supplement.

**Table 5.** Safety.

	Baseline to week 16 (n = 100)	Weeks 16–28		Weeks 28–52 (n = 100)	Baseline to week 52 (n = 100)
		Monotherapy (n = 16)	Combination therapy (n = 76)*		
Any TEAE	75 (75)	6 (38)	53 (70)	2 (2)	89 (89)
TEAE by severity					
Mild	20 (20)	4 (25)	22 (29)	0 (0)	23 (23)
Moderate	41 (41)	1 (6)	16 (21)	2 (2)	41 (41)
Severe	12 (12)	1 (6)	11 (14)	0 (0)	19 (19)
Serious	2 (2)	0 (0)	4 (5)	0 (0)	6 (6)
TEAE by relationship to study drug					
Not related	45 (45)	5 (31)	30 (39)	1 (1)	43 (43)
Related	30 (30)	1 (6)	23 (30)	1 (1)	46 (46)
Serious TEAE	18 (18)	1 (6)	16 (21)	0 (0)	30 (30)
Death	2 (2)	0 (0)	1 (1)	0 (0)	3 (3)

Data are number of patients (%).

* Eight patients discontinued before week 16 (when determination of monotherapy or combination therapy occurred) and were excluded from week 28 analysis.

TEAE, treatment-emergent adverse event.

## Discussion

In the COMPASS-3 study, we evaluated whether 6MWD as a solitary treatment target was clinically meaningful and appropriate for the design of a clinical trial. Overall, 31 patients in the ITT population achieved the primary endpoint. Patients who did not reach 6MWD threshold at week 16 were more likely to be women, at WHO FC III or IV, and have a shorter 6MWD at baseline. Counterintuitively, patients who did not reach 6MWD threshold at week 16 had more preserved hemodynamics at baseline. One potential explanation of better hemodynamics seen in patients who did not reach 6MWD threshold is the MRI data showing less adaptive and more maladaptive RV remodeling. For instance, there was a moderate inverse correlation between RVEDV/LVEDV ratio and 6MWD at baseline (r = −0.541) for patients who experienced clinical worsening and/or decline, but a positive correlation for patients without clinical worsening and/or decline. Thus, RV dilation is linked to worse functional capacity in patients who did poorly clinically (maladaptive remodeling) while in clinically stable patients RV dilation was associated with better functional capacity (adaptive remodeling).

Importantly, achieving 6MWD threshold at either 16 or 28 weeks failed to predict clinical outcome. This observation, combined with aforementioned discordance among individual risk components and outcome within individual PAH patients strongly suggest that clinicians should make use of a wide range of risk factors, as opposed to one or two, when accessing overall risk and treatment response in any individual patient.^14,15^ These data also exemplify the need to go beyond the use of general risk profiles, as suggested in recent guidelines,^9^ as patients may exhibit risk features that span across these individual risk profiles. The use of stratified risk equations or calculators, as described in contemporary literature,^14^ may balance these non-weighted siloes of risk leading to better prediction of outcome for any individual patient.

It is important to note that while up-to-date guidelines were followed when this study was designed in 2007, the current standard of care has since changed. At the time the trial was designed, a treat-to-target approach was recommended,^31,32^ which was recently updated to emphasize a multivalent treatment approach which reduces patient mortality risk.^9^ In our analysis, patients in the monotherapy group achieved a greater 6MWD at all time-points compared with patients treated with combination therapy. In addition, the 6MWD between 16 and 28 weeks did not change dramatically in the combination therapy group (299 vs. 309 m) (Table S3). This implies that addition of sildenafil to bosentan did not impact 6MWD, and suggests a lack of overall efficacy on this parameter. While 6MWD may correlate with patient outcome, its use as the solitary endpoint in this clinical trial was not beneficial. Future clinical trials should consider the use of composite risk scores as potential endpoints to produce more clinically meaningful results.

Current guidelines recommend upfront or sequential combination therapy in order to target multiple PAH disease pathways.^9^ In this study, combination therapy consisted of the recommended (in the respective prescribing information) doses of bosentan and sildenafil. However, both this study and the results from the recently published long-term outcome COMPASS-2 study (which both missed their endpoints) suggests that the combination of sildenafil with bosentan may not be effective.^33^ In combination, sildenafil efficacy may have been reduced, since bosentan reduces the plasma concentration of sildenafil by approximately 50%.^34^ More recently, data from the phase 3 AMBITION trial demonstrated the clinical benefit of ambrisentan + tadalafil (another phosphodiesterase type 5 inhibitor) in patients with PAH.^35^ Additionally, the clinical effectiveness macitentan in the SERAPHIN phase 3 clinical trial was reported in both treatment-naïve patients and in patients receiving background therapy with a PDE5i and/or inhaled prostanoids.^36^ Further supporting the use of combination therapy, the GRIPHON phase 3 clinical trial evaluated the effectiveness of the oral IP receptor agonist selexipag in both treatment-naïve patients and in patients receiving background therapy.^37^

Bosentan-based therapy led to improvements in other meaningful parameters, including WHO FC, neurohormone levels, and hemodynamics. There were significant positive changes in RV geometry, function, and LV relationships that resulted in improved parameters of left ventricle filling (LVEDV and PAWP) and correlated with clinical outcome. Correlations between cMRI-derived and RHC-derived measures of cardiac function at baseline also suggest the potential for cMRI to serve as an alternative, or adjunct, to RHC in assessing functional derangements, patient stability, and need for sequential or upfront combination therapy. Similar to transthoracic echocardiography (TTE), cMRI is non-invasive, but offers higher resolution, more precise measurements, and greater reproducibility than TTE.^38,39^ In clinical practice, tricuspid annular plane systolic excursion, right ventricular fractional area change, systolic velocity, and RV global strain are recommended in follow-up visits for patients with PAH. Although optimal timing is currently unknown, structural changes to the heart can be observed using cMRI or TTE within 4–6 months. While cMRI may not be practical or cost-effective in regular clinical practice, it may provide better data in clinical trials. In addition, these data further confirm previous studies demonstrating the benefits of TTE in clinical trials. In the BREATHE-1 TTE sub-study of 85 patients with PAH, data showed improved TTE variables (e.g. RV systolic function and increase in LV size) in patients treated with bosentan compared with placebo.^40^

The additional and clinically appropriate relationships between neurohormone levels and 6MWD and measures of RV remodeling by MRI further support the adjunctive role of MRI in accurately characterizing severity of illness in this patient population.

The percentage of patients who experienced clinical worsening and/or decline at week 52 was higher in the combination therapy group compared with the monotherapy group and could reflect that RV remodeling was more adaptive in patients that did not have a clinical worsening event. Additionally, 6MWD has been shown to be prognostic and the monotherapy group had significantly higher 6MWD at baseline compared with the combination therapy group. Further, analysis using the REVEAL risk score calculator showed that at week 16, when patients were divided into groups based on their 6MWD, patients in the combination therapy group had a higher risk score compared to patients in the monotherapy group, reflecting a greater propensity for future events.

Interestingly, time to clinical worsening and/or decline did not differ between patients who did or did not achieve 6MWD threshold at week 16. However, lower RVEF and LVEDV index, RV/LV systolic and diastolic ratios and higher RV mass index at baseline were associated with clinical worsening and/or decline, which extends previous MRI findings related to mortality in patients with PAH.^24,27^ In addition, the observation that reverse RV remodeling contributed to the prediction of clinical outcome further supports the use of serial MRI as a management tool in PAH. Combining these changes with those seen in hemodynamics or other clinical parameters, such as NT-pro-BNP expression, support the use of a multimodality model in guiding therapeutic choices in these critically ill patients.

Univariable models showed that many cMRI variables were predictive of clinical worsening or decline. However, multivariable analysis showed that many of these cMRI parameters were closely associated and determining individual effects was not statistically feasible. The multivariable model that included RVEDV at baseline and change in PVR from baseline to week 16 best predicted clinical worsening and/or decline. The need to consider multiple endpoints to predict outcome in patients with PAH is not without precedent. In the French PAH registry, sex, baseline 6MWD, and cardiac output were jointly associated with three-year survival.^41^ Similarly, in the REVEAL registry, numerous parameters, including PVR, PAH etiology, and WHO FC, were collectively predictive of one-year survival.^8^ The French and REVEAL multivariable models were subsequently validated in prospective cohorts of patients with PAH.^14,42^ The COMPASS-3 multivariable model requires validation in a larger cohort as it was generated post-hoc in a limited number of patients.

In terms of limitations, COMPASS-3 was a phase 4 open-label study and therefore not a randomized controlled study, and some of the reported analyses were conducted post-hoc. Despite these caveats, the data reported herein represent the most complete set of hemodynamic and cMRI-derived data from patients with PAH published to date. There were 17 (17%) clinical worsening/decline events over the one year of follow-up and this low event rate greatly limits post-hoc comparisons of sequential predictors of worsening. These results may also contain possible bias as the baseline mean 6MWD may be skewed lower due to the inclusion criteria of 6MWD of 150 m. It should be noted that the bioavailability of both sildenafil and bosentan are altered when used in combination.^43^ In a study of 125 patients with PAH, combinations of sildenafil and bosentan led to a significant decrease in the bioavailability of sildenafil compared to combinations of macitentan and bosentan (*P* < 0.001) whereas bosentan concentrations were greatly increased in patients when combined with sildenafil. Therefore, the findings of combination therapy in this study may be unique to combinations of sildenafil and bosentan.

In conclusion, using a singular endpoint (6MWD > 380 m) did not serve as a clinically meaningful prognostic indicator and our analyses indicate that a more comprehensive assessment of risk is needed. We detected moderate-to-strong correlations between cMRI-derived and RHC-associated parameters of cardiac function and found cMRI to be both prognostic of clinical outcome and sufficiently sensitive to detect reverse RV remodeling.
